# Continuing evidence of Chagas disease along the Texas-Mexico border

**DOI:** 10.1371/journal.pntd.0006899

**Published:** 2018-11-14

**Authors:** Melissa S. Nolan, David Aguilar, Eric L. Brown, Sarah M. Gunter, Shannon E. Ronca, Craig L. Hanis, Kristy O. Murray

**Affiliations:** 1 Department of Pediatric Tropical Medicine, Baylor College of Medicine, Houston, TX, United States of America; 2 Department of Epidemiology, Human Genetics, and Environmental Sciences, University of Texas Health Science Center at Houston, Houston, TX, United States of America; 3 Department of Cardiology, Baylor College of Medicine, Houston, TX, United States of America; Universidad del Valle de Guatemala, GUATEMALA

## Abstract

**Background:**

Chagas disease is a chronic parasitic infection that progresses to dilated cardiomyopathy in 30% of human cases. Public health efforts target diagnosing asymptomatic cases, as therapeutic efficacy diminishes as irreversible tissue damage progresses. Physician diagnosis of Chagas disease cases in the United States is low, partially due to lack of awareness of the potential burden in the United States.

**Methodology/Principal findings:**

The current study tested a patient cohort of 1,196 Starr County, Texas residents using the Hemagen Chagas ELISA Kit as a preliminary screening assay. Samples testing positive using the Hemagen test were subjected to additional confirmatory tests. Two patients (0.17%) without previous Chagas disease diagnosis were identified; both had evidence of acquiring disease in the United States or along the Texas-Mexico border.

**Conclusions/Significance:**

The Texas-Mexico border is a foci of Chagas disease human cases, with a local disease burden potentially twice the national estimate of Hispanic populations. It is imperative that physicians consider persons with residential histories along the Texas-Mexico border for Chagas disease testing.

## Author summary

Chagas disease is a parasitic infection, which in 30% of people results in chronic dilated organomegaly over the course of decades. This progressive disease typically advances subclinically until end stage heart failure is established, at which point treatment is no longer efficacious. Thus, current public health efforts target screening of high-risk populations for early disease detection and clinical management for improved health outcomes. Although historical evidence suggests the Texas-Mexico border as a region with high Chagas disease prevalence, surveillance is nonexistent. Our study indicates this region likely has an increased risk for undiagnosed morbidity. Furthermore, occupational histories were highlighted as key risk factors for both cases in this report. Physicians should consider Chagas screening in persons with residential histories along the Texas-Mexico border.

## Introduction

Chagas disease is caused by infection with the protozoan parasite *Trypanosoma cruzi*. Primarily transmitted by infected triatomine vector species in the Americas, congenital transmission, blood transfusion, organ transplantation, or ingestion of contaminated beverage/food products are also known to contribute to human disease. The estimated global disease burden is over 6 million [1], with up to 30% of infected patients experiencing clinical symptoms as a result of infection [2,3]. Over the course of decades, clinical disease manifests as progressive cardiac or gastrointestinal tissue dilation. Early identification of these clinical cases is critical, as the presence of irreversible tissue damage is inversely proportional to drug efficacy [4] and survival [5].

Eleven triatomine species exist in the United States–[6] with historical reports of these species dating back to the early 1800s [7]. Sylvatic transmission cycles, with raccoons, opossums, woodrats and dogs serving as important mammalian reservoirs have been reported in seventeen southern states [8]. Despite continual documentation of *T*. *cruzi* positive triatomines and animal reservoir species in the United States [6,9], autochthonous human Chagas disease cases have been rarely described. Of the six states with published evidence of autochthonous vector-borne transmission to humans (n = 43 total cases) (Arizona, California, Louisiana, Mississippi, Tennessee, and Texas), Texas is home to a disproportionate burden of reported cases [6, 10–14].

Specifically, the Rio Grande Valley region has been identified as a hot-spot of positive patient populations based on historical evidence demonstrating that in 1980, 2.4% of residents screened positive for Chagas [15]. This Texas-Mexico border region (Cameron, Willacy, Hidalgo, and Starr counties) has long been theorized to be a high-risk area for vector-borne disease due to pervasive poverty, substandard housing, barriers to healthcare access, and weaknesses in the public health infrastructure [16]. Renewed efforts of Chagas disease surveillance in the state have peaked interest in understanding the contemporary disease burden in this potentially high-risk community. A recent screening of banked sera from Cameron County, Texas revealed that 0.36% of residents had a confirmed Chagas disease infection [12]. With each colonia having different and unique social determinants of health [17], our current study aimed to continue surveillance in neighboring Starr County and to further understand the Chagas disease prevalence and risk factors for infection in this local population.

## Materials and methods

In February 1981 the Starr County Health Studies program was established by investigators from the University of Texas Health Science Center at Houston with the opening of a field office in Rio Grande City, Texas. Since then and continuing today, a series of studies has been conducted to understand the genetics and epidemiology of type 2 diabetes, its complications and related conditions. In total, some 10,000 local residents have participated in more than 28,000 examinations generating more than 500,000 banked biological samples [18].

For the purposes of our current investigation, banked sera from 1,196 study participants were available for testing from study of type 2 diabetes, sleep apnea, and endothelial function [19]. All samples were initially screened by ELISA using Hemagen Chagas Kit (Hemagen Diagnostics Inc, Columbia, MD). Any samples positive by Hemagen Chagas Kit were further tested using Chagas STAT-PAK assay (Chembio Diagnostic Systems Inc, Medford, NY) and Chagas DPP assay (Chembio Diagnostic Systems Inc, Medford, NY). Lastly, any sample positive by one or more of the described tests was sent to the US Centers for Disease Control (CDC) Parasitic Disease Branch for confirmation testing: Weiner EIA and trypomastigote excreted-secreted antigens (TESA) immunoblot. Confirmed positives were defined as being positive on at least one study assay and at least one CDC confirmation assay. Confirmed positives were invited to take part in a follow-up examination, comprised of a medical chart abstraction, a contemporary health survey, and electrocardiogram (ECG).

Protocols were approved by institutional review boards at the University of Texas Health Science Center at Houston (HSC-SPH-02-042) and Baylor College of Medicine (H-35471), respectively. Follow-up evaluation of Chagas seropositive patients was performed through enrollment under Baylor College of Medicine (BCM)’s protocol. All adult subjects provided informed written consent.

## Results

From 2010 to 2014, 1,196 Starr County residents were enrolled in a comprehensive examination that included anthropometric evaluations, electrocardiography (ECG), echocardiography (ECHO), medical and medication histories, an in-home overnight sleep evaluation, end evaluation of endothelial function and aortic stiffness (19). Fasting plasma, serum and urine specimens were obtained from all individuals while those with no previous diagnosis of diabetes having an oral glucose tolerance test and those with type 2 diabetes having a standard meal challenge. Of the 1,196, 602 were classified as having type 2 diabetes. Complete details of the sampling and disease burden are in Hanis et al. (2016) [19].

**Fig 1:**
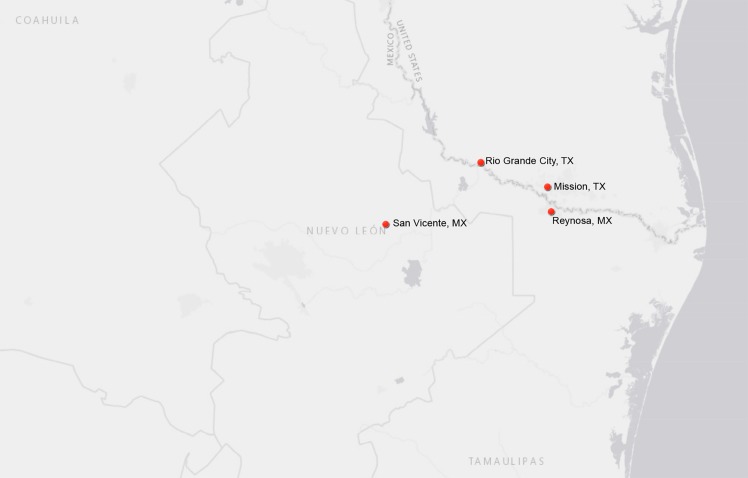
Location of study site and important locations of Chagas positive participants. This figure was generated in ArcGIS. The authors purchased the software license and own rights to the figure. Additionally, this figure was approved for publication using PACE.

Participants (n = 1,196) previously enrolled were tested for Chagas and eight screened positive by Hemagen Chagas Kit (Table 1). Of these eight individuals, one was positive by Chagas STAT-PAK assay, two were positive by DPP, two were positive by Weiner EIA, and one was positive by TESA immunoblot. Per our diagnosis criteria, we determined that two participants (Sample IDs #4788 and #5070) were Chagas positive. The follow-up risk factor analysis, clinical assessments, and locations of importance (Fig 1) are listed below.

**Table 1:** Discordant testing results among Chagas disease diagnostics.

Sample ID	Baylor College of Medicine testing			US CDC testing	
	Hemagen	Stat-Pak	DPP	Weiner EIA	TESA
4029	+	-	-	0.000	
4052	+	-	-	0.004	
4202	+	-	-	0.009	
4287	+	-	-	0.000	
4657	+	-	-	0.052	
4788	+	-	+	**0.546**	Negative
5070	+	+	+	**1.293**	**Positive**
5187	+	-	-	0.000	
Weiner Results Key Neg < 0.270 Pos >0.330 Ind 0.270–0.330					

Case-patient 1 was a 76-year old Hispanic female with a 43-year history of diabetes mellitus and 20-year history of hypertension. On May 11, 2017, she tested positive for Chagas antibodies on Hemagen Chagas Kit, DPP and CDC Weiner EIA, and negative for Chagas antibodies on STAT-PAK and CDC TESA immunoblot. Her ECG on August 23, 2017 indicated the presence of a 1^st^ atrioventricular block and left ventricular hypertrophy with repolarization abnormality. She reported a history of an “enlarged heart” but was unable to provide additional clinical details at the time of assessment nor did she complete an echocardiogram. While she regarded her medical management as acceptable, her clinical complaints at the time of follow-up were continued pedal edema and inability to climb two flights of stairs.

She was born in Mission, Texas where she has lived her whole life with the exception of 15 years during her early adulthood when she lived in Bartow and Winter Haven, Florida. Case-patient 1 was a mother to four children, now all adults, who were unavailable or uninterested in Chagas testing. She reported no travel to Latin American countries except for infrequent shopping day trips at the border town of Reynosa, Mexico. Her risk factors for acquiring Chagas included possible congenital transmission from her mother (born, raised, and lived in Nuevo Leon, Mexico) and occupational exposures. As a child, she reported frequently performing migrant work in Tennessee, west Texas, California, Iowa, and Colorado. She reported staying in “shacks” while working, but had never seen triatomine insects. Based on her discordant testing results, indicating a low antibody titer, we theorize that transmission occurred earlier in her life, in either Texas or a southern state in which she previously worked. This is not surprising since both quantitative (decrease in the number of antibody-secreting B-cells) and qualitative (activity of different B-cell subsets including changes to the specificity of the antibody repertoire) changes are associated with aging [20]. More specifically, decrease in IgG titers with specificity to vaccine antigens have been described [21].The exact timing cannot be determined based on her lack of triatomine recognition and reported lack of triatomine exposure.

Case-patient 2 was a 35-year old Hispanic male with an unremarkable health history. On May 11, 2017, he tested positive on all three in-house tests and both of CDC’s confirmatory tests. At his follow-up appointment on August 23, 2017, his ECG was normal and he had no clinical complaints. He was born in Rio Grande City, Texas, where he resided for 25 years before moving to Alabama for 2 years, and then returning back to Rio Grande City, Texas. From the ages of 17–25, he split his time between Rio Grande City, Texas and San Vicente, Nuevo Leon, Mexico (74 miles away). His mother and maternal grandmother were both from Nuevo Leon, Mexico, posing the potential for congenital transmission.

His family has had ranches in Rio Grande City and San Vicente where he has worked as his primary occupation for over 20 years. His job responsibilities have included fencing, feeding animals, clearing land, and gardening. The family ranch in Mexico has had collective animal housing (goats, chickens, cows, lambs, and goats), where he reported “many times” seeing triatomines in the animal bedding. He also reported seeing triatomines in Rio Grande City on the trees outside his home. He never recalled seeing triatomines inside his home at either location. Given his extensive history of working in areas endemic to triatomines, he likely acquired infection at one of the two residential locations.

## Discussion

Chagas disease is a significant public health threat in numerous parts of the world. It is mainly considered a tropical disease and has received limited attention in the United States. This study adds to the growing body of work establishing Chagas disease as a considerable public health concern along the Texas-Mexico border. Owing to the significant morbidity among those infected, there should be an increased awareness in endemic triatomine areas and increased testing among those with pathologies consistent with infection such as unexplained heart failure. Historical evidence of autochthonous transmission in the state dates back to 1955 and surveillance studies have continually demonstrated high disease burdens among Hispanic foreign-born populations [22]. Despite clear evidence presented here and in our earlier screen in Cameron County [12] that Chagas continues to be present, there is no unified public health set of guidelines for its screening in either the general or specialized clinic population. Consequently, Chagas disease has long been regarded as the most neglected of the neglected tropical diseases [23], and scientific literature suggest that this ‘neglect’ extends to some regions along the Mexico-United States border [24–26]. Lack of awareness by physicians, barriers in healthcare access, a paucity of efficacious treatment options, and substandard public health resources continue to contribute to preventable morbidity and mortality due to Chagas disease nationally [27].

It is critical we clarify the disease burden and epidemiology of transmission to improve physician awareness. An important component of developing enhanced patient profiles for physician education is identifying foci of vector-borne transmission and elevated population disease burdens. Our study demonstrated that Chagas disease infections in Starr County, Texas are higher than the estimated prevalence among foreign-born Hispanic United States residents [0.17% (2/1,196) vs. 0.09% (300,000/323,000,000[28]), respectively]. Furthermore, our study is consistent with a previous study which found 0.36% infection prevalence from human residents sampled from 2005–2008 in neighboring Cameron County, Texas [12]. Similarly, the border state of Nuevo Leon, Mexico has a documented history of autochthonous transmission, with a recent study in 2014 identifying a 2% seroprevalence among residents [2] and a 2017 study identifying 14.5% of sylvatic animals seropositive [29].

An important limitation to our study is our inability to determine the precise incident of transmission. This limitation is inherent due to our serologic diagnostic method of this life-long infection; however, based on the patients’ epidemiologic risk profiles and relatively limited travel histories, the data suggest that both patients likely acquired their infections in the United States or along the Texas-Mexico border. An implication was our finding of discordant testing results, indicating a lack of specificity of commercially available diagnostic assays for diagnosing strains of *T*. *cruzi* that evolved in North America compared to South American strains. Chagas disease diagnostics have been suboptimal [30] and our study highlights that Texas-Mexico border patient populations present similar point-of-care testing challenges.

Chagas disease is a neglected tropical disease in the United States. Our research adds to the growing body of evidence that this disease is likely endemic along the Texas-Mexico border with infection prevalence higher than that of the overall foreign-born Hispanic United States resident population. However, due to limitations in the sample size for this study, it is difficult to make assumptions regarding larger populations. Physicians should be aware of the potential elevated disease risk among this geographic patient population.
